# The Effect of Mutation in Lipopolysaccharide Biosynthesis on Bacterial Fitness

**DOI:** 10.3390/cells11203249

**Published:** 2022-10-16

**Authors:** Laura Nagy, Péter Urbán, Lilla Makszin, Viktor Sándor, Anikó Kilár, Hajnalka Ábrahám, Beáta Albert, Béla Kocsis, Ferenc Kilár

**Affiliations:** 1 Institute of Bioanalysis, Medical School, University of Pécs, Szigeti út 12, 7624 Pécs, Hungary; 2 Szentágothai Research Center, University of Pécs, Ifjúság útja 20, 7624 Pécs, Hungary; 3 Department of Medical Biology and Central Electron Microscope Laboratory, Medical School, University of Pécs, Szigeti út 12, 7624 Pécs, Hungary; 4 Department of Bioengineering, Sapientia Hungarian University of Transylvania, Libertăţii Sq. 1, 530104 Miercurea Ciuc, Romania; 5 Department of Medical Microbiology and Immunology, Medical School, University of Pécs, Szigeti út 12, 7624 Pécs, Hungary

**Keywords:** lipopolysaccharides, *Shigella sonnei* 4351, lipopolysaccharide biosynthesis, bacterial genome, mutation, endotoxin

## Abstract

This paper presents the genome sequence of a *Shigella sonnei* mutant strain (*S. sonnei* 4351) and the effect of mutation in lipopolysaccharide biosynthesis on bacterial fitness. Lipopolysaccharides are the major component of the outer leaflet of the Gram-negative outer membrane. We report here a frameshift mutation of the gene *gmhD* in the genome of *S. sonnei* 4351. The mutation results in a lack of epimerization of the core heptose while we also found increased thermosensitivity, abnormal cell division, and increased susceptibility to erythromycin and cefalexin compared to the *S. sonnei* 4303. Comparative genomic analysis supplemented with structural data helps us to understand the effect of specific mutations on the virulence of the bacteria and may provide an opportunity to study the effect of short lipopolysaccharides.

## 1. Introduction

Lipopolysaccharides (LPSs) are the most abundant macromolecules on Gram-negative bacterial surfaces and consist of a hydrophobic lipid A section, a hydrophilic core oligosaccharide, and a hydrophilic O side-chain. The LPSs without the oligosaccharide chains are the rough lipopolysaccharides (R-LPSs) and the complete structures are the smooth lipopolysaccharides (S-LPSs). The composition and structural variabilities of the LPSs highly influence the biofilm-forming ability and the pathophysiological impact of Gram-negative bacteria. Generally, bacteria with defective and short LPSs are more sensitive to hydrophobic antibiotics; therefore, the study of inhibitors of LPS biosynthesis provides a good opportunity to develop antibiotic adjuvants and antimicrobial agents. However, only a limited number of inhibitory substances are available with known effects.

**Figure 1:**
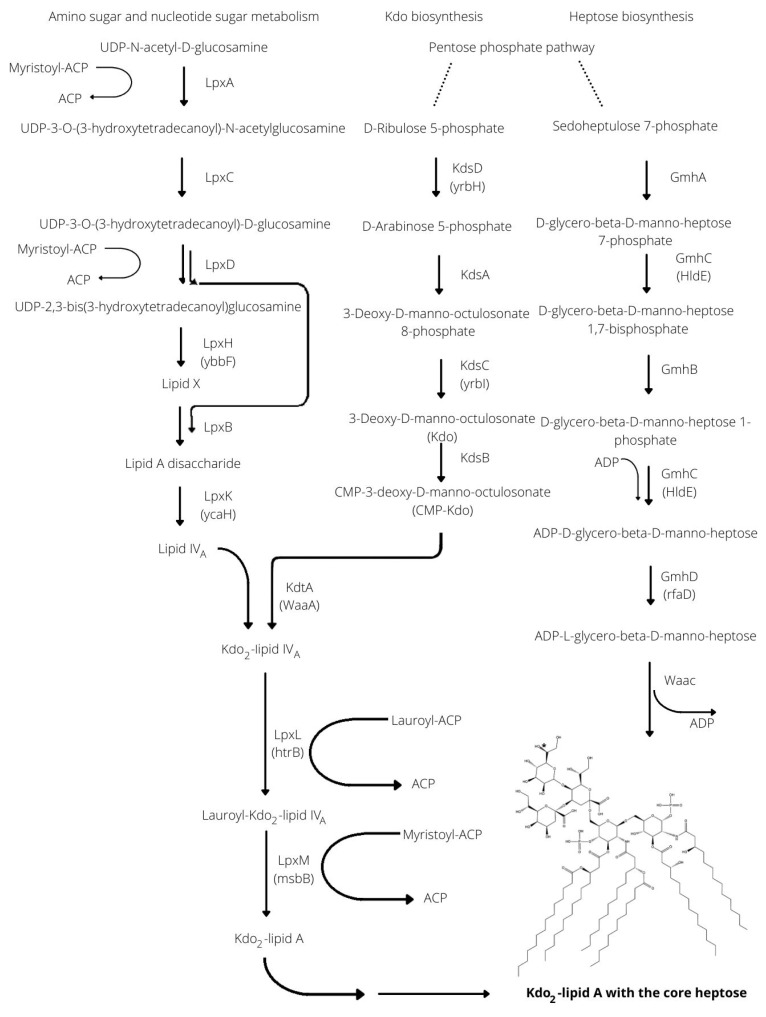
The scheme showing the synthesis steps of the three major constituents—Lipid IV_A_, Kdo, and ADP-L-glycero-β-D-manno-heptose—of the Rd2-LPS structure. The enzymes involved in the biosynthetic steps are indicated at the arrows. The star (*) is labeling the D configuration of the OH-group in the heptose structure.

The biosynthesis of the lipopolysaccharides includes the separate syntheses of the major constituents of lipid A: the tetra-acylated lipid A precursor (Lipid IV_A_), 2-keto 3-deoxy-D-manno-octulosonate (Kdo) sugars, and the heptose (Hep) sugars [1]. The scheme in detail is seen in Figure 1, showing the major enzymes involved in the synthetic pathway.

The first step in the biosynthesis of Lipid IV_A_ is the acetylation of UDP-N-acetyl-α-D-glucosamine with β-hydroxymyristoyl-ACP by the LpxA enzyme [2], followed by deacetylation with LpxC [3]. The LpxD [4] enzyme incorporates a second hydroxyl-myristate into the structure and then, the LpxH [5] cleaves the UDP moiety, resulting in a so-called lipid X molecule. The LpxB [6] then forms the lipid A disaccharide from a lipid X and a UDP-2,3-bis(3-hydroxytetradecanoyl) glucosamine. The 4’ position of the lipid A disaccharide is phosphorylated by LpxK [7], resulting in the Lipid IV_A_.

The Kdo molecules are formed from D-ribulose 5-phosphates in consecutive enzymatic conversions by the KdsD isomerase [8], the Kdo 8-phosphate synthase (KdsA) [9], and the Kdo 8-phosphatase (KdsC) [10]. After forming a CMP-Kdo by the KdsB synthase [11], two CMP-Kdo are used consecutively by the KdtA [12] enzymes to couple two Kdo parts to form the Kdo2-lipid IVA. The next steps are the binding of laurate (catalyzed by the acyltransferase LpxL) [13→7] and myristate (catalyzed by LpxM) [13] acyl chains, resulting in six acyl chains in the structure.

The heptose biosynthesis [14] involves the sedoheptulose-7-phosphate, an intermediate of the pentose phosphate pathway. The GmhA [15] enzyme catalyzes the isomerization of the molecule to D-glycero-β-D-manno-heptose-7-phosphate. This heptose is phosphorylated by the bifunctional GmhC [14] enzyme, resulting in a D-glycero-β-D-manno-heptose-1,7-bisphosphate. The next step is the phosphate removal from the C-7 position by the GmhB phosphatase [14] and then, an ADP is bound to D-glycero-β-D-manno-heptose-1-phosphate by the GmhC (the second function of this enzyme). In some bacteria, two separate proteins (the HldA kinase and the HldC, which is an ADP-transferase) carry out the two functions of GmhC [14,16]. The chiral configuration of the ADP-heptose is changed by the isomerase GmhD [17] (formerly named RfaD) to yield ADP-L-glycero-β-D-manno-heptose molecules by epimerization.

The Waac enzyme [18] creates the final minimal structure of the lipopolysaccharides from an ADP-L-glycero-β-D-manno-heptose and the Kdo2-Lipid IV_A_. While most bacteria have all three of the above-described constituents, there are some exceptions. Bacteria may remain viable without the core heptoses and even Kdo sugars. Nonetheless, those strains lacking Kdo are not able to thrive within their native niche; however, those constructions were only designed in laboratories [1].

Antibiotic resistant bacteria, particularly multi-drug resistant strains, are a growing crisis worldwide, especially in medical facilities. Because of the problem of cross-resistance, the main goal is to find new cellular targets in order to inhibit structural changes (e.g., in lipopolysaccharides) due to environmental effects, thereby reducing bacterial fitness. The most promising LPS biogenesis inhibitors are compounds that target the LpxC enzyme [19,20,21,22]. The best candidates as targets could be the GmhA and the GmhD enzymes, being part of the biosynthetic pathway of the heptose sugar. The inhibition of GmhB is not recommended as it results in the accumulation of D-glycero-D-manno-heptose 1,7-bisphosphate, a molecule suggested recently as a pathogen-associated molecular pattern [23]. Lipopolysaccharide research in vaccine development is also important since lipopolysaccharides specifically target the immune system [24,25,26].

This project aimed at creating a reference genome to elucidate the genetic background of known LPS mutant phenotypes [27] and better understanding the LPS biosynthesis by means of comparative genomic analysis supplemented with LPS structural data and detailed phenotypic study.

## 2. Materials and Methods

### 2.1. Strains and Storage

The *Shigella sonnei* strain was isolated in Pécs [28] and a lipopolysaccharide mutant line was generated by random mutagenesis, with ethyl methanesulfonate causing plasmid loss during passages [29]. Previous studies described the LPS structures in the mutant line, including the Phase II *S. sonnei* (also called *S. sonnei* 4303) considered as the mother strain, the *S. sonnei* 4351 (formerly named *S.* *sonnei* 562H) containing D-glycero-D-manno-heptose (D,D-heptose) instead of L-glycero-D-manno-heptose (L,D-heptose) in the core structure, and the completely heptoseless *S. sonnei* 4350 [27]. From these strains and the *Salmonella minnesota* R595 mutant, we could isolate ADP-D-glycero-D-mannoheptose and ADP-L-glycero-D-mannoheptose, which are intermediers in the biosynthesis of the heptose components in the core structures [30,31,32,33]. The *S. sonnei* strains formerly were freeze-dried; nowadays, they are frozen with liquid nitrogen, with the addition of 38% glycerol and stored at −80 °C.

### 2.2. Sequencing

Isolation and genomic library preparation of *S. sonnei* 4351 was performed as described previously [34], employing the Qiagen DNeasy Plant Mini Kit (Qiagen, Germantown, MD, USA), the Ion Xpress Plus Fragment Library Kit, and the Ion 316 Chip with the Ion Torrent PGM sequencer (Thermo Fisher Scientific Inc., Waltham, MA, USA) according to the recommendation of the manufacturer. Whole transcriptome sequencing was also performed using Ion Torrent Ion Total RNA-Seq Kit v2 (Thermo Fisher Scientific Inc., Waltham, MA, USA) according to the manufacturer’s recommendation.

### 2.3. Genomic Analysis

The genomes of *S. sonnei* Ss046 and *S. sonnei* 53G were used for reference. The only *S. sonnei* strain with a whole lipopolysaccharide pathway is *S. sonnei* Ss046 presented in the Kyoto Encyclopedia of Genes and Genomes database [35]. The closest relative of *S. sonnei* 4303 is *S. sonnei* 53G, according to Clustal Omega analysis with 16S rRNA and 5 housekeeping genes (*adk*, *fumC*, *gyrB*, *mdh*, *purA*) [34]. Comparative analysis was performed with *S. sonnei* 4303, also referred to as phase II *S. sonnei*, being the mother strain of the LPS mutant line [34]. The de novo assembly of the genome of *S. sonnei* 4351 was performed using the SPAdes v3.1 Genome Assembler software [36]. The whole-genome alignment was made in the Mauve software [37] with default parameters, where scaffolds in the draft assemblies were reordered to a reference genome (*S. sonnei* 53G). The Prokka v. 1.9 software was applied to perform sequence annotation [38]. The *S. sonnei* 4303 data were used to scaffold the contigs of *S. sonnei* 4351. The whole genome sequence of *S. sonnei* 4351 was deposited in the GenBank under the accession number PRJNA400697 [39].

Gene expression analysis was performed using Geneious 8.1. (https://www.geneious.com (accessed on 1 June 2016)). Genes involved in the lipopolysaccharide biosynthesis were examined with the BlastN—basic local alignment search tool by searching in the nucleotide collection (nr/nt) database using Megablast [40]. The nomenclature of the LPS genes were used according to the Kyoto Encyclopedia of Genes and Genomes database [35].

### 2.4. Electron Microscopy

The samples were prepared to perform scanning electron microscopy analysis by dewatering with acetone series on slides. Images were created by a JSM 6300 Scanning electron microscope (JEOL, Akishima, Tokyo, Japan).

### 2.5. Measuring Antibiotic Susceptibility

The minimal inhibitory concentration (MIC) was determined by the tube dilution method [41].

### 2.6. Testing Thermosensitivity

The growth of the bacteria at different temperatures was monitored in the range of 37–45 °C on LB (Luria–Bertani) agar plates. The growth of *S. sonnei* 4351 was compared to that of *S.* *sonnei* 4303.

### 2.7. qPCR

RNA extraction was performed using liquid nitrogen and the NucleoSpin RNA kit (Takara Bio, San Jose, CA, USA). The cDNA was transcribed with a high-capacity cDNA reverse transcription Kit (Thermo Fisher Scientific Inc., Waltham, MA, USA) using the StepOne Plus (Thermo Fisher Scientific Inc., Waltham, MA, USA), the TaqMan PCR master mix (Thermo Fisher Scientific Inc., Waltham, MA, USA), and the TaqMan Mini Kit (IDT, Coralville, Iowa, USA). UiaD (beta-glucuronidase) was used as the endogenous control. The TaqMan qPCR primers and probes used in the analysis are given in Table 1. Fold change, describing the gene expression difference, was calculated using the delta–delta Ct method: fold change = 2^−ΔΔCt^, where Ct is the measured cycle threshold.

### 2.8. Sanger Sequencing

To validate the findings of the Ion Torrent sequencing on mutation detected for *gmhD*, Sanger sequencing was performed using the ABI PRISM 310 Genetic Analyzer (Thermo Fisher Scientific Inc., Waltham, MA, USA) and the BigDye v3.1 reagents (Thermo Fisher Scientific Inc., Waltham, MA, USA). Table 2 shows the primer sequences to amplify the *gmhD* gene.

### 2.9. 3D Protein Structure Prediction

The 3D protein structures of the *gmhD* gene products were created by the AlphaFold multimer software (EMBL-EBI) [42]. The per-residue confidence score (pLDDT) calculated by the AlphaFold method ranges from 0 to 100, where pLDDT values above 90 are considered to be high accuracy. The confidence values were pLDDT = 95.9 for *Shigella sonnei* 4303 and pLDDT = 90.3 for *Shigella sonnei* 4351.

## 3. Results

We determined the 4.57 Mb long genome sequence of *Shigella sonnei* 4351 and deposited in the GenBank under the accession number PRJNA400697 [39]. The complete genome contains 4607 protein-encoding sequences, 68 tRNA genes, 10 rRNA genes, and a CRISPR region. *S. sonnei* 4351 is a member of a mutant line series examined in several previous lipopolysaccharidomic studies [29,30,31]. Comparative genomics analyses were performed to compare the genomes of the *S. sonnei* 4351; the *S. sonnei* 4303 (the mother strain); and the two reference strains, *S. sonnei* Ss046 and *S. sonnei* 53G, respectively.

By analyzing the genes of the lipid A synthetic pathway in the *S. sonnei* 4303 and in the *S. sonnei* 4351, we found that the genes, *lpxA, lpxC, lpxD, lpxH, lpxB, and lpxK*, were identical to the genes known in the *S. sonnei* Ss046 reference strain. The genes of the KdsD, KdsC, KdsB, KdtA, LpxL, and LpxM proteins in both strains (*S. sonnei* 4303 and 4351) were 100% identical to those in the *S. sonnei* Ss046. The genomic sequences of the KdsA enzyme in the *S. sonnei* 4303 and *S. sonnei* 4351 species showed polymorphism to the corresponding gene in the *S. sonnei* Ss046; however, they were identical to the sequence in the *S. sonnei* 53G.

**Figure 2:**
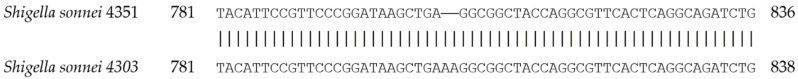
The nucleotide sequences of the *gmhD* genes of *S. sonnei* 4351 and *S. sonnei* 4303, respectively, aligned from the 781 position. The alignment shows a gap in the sequence of *S. sonnei* 4351 at positions 806 and 807, leading to a frameshift mutation of the *gmhD* gene in *S. sonnei* 4351 and thus, a smaller predicted gene product.

Analyzing the genes related to the heptose biosynthesis, 100% matches to the corresponding genes of GmhA, GmhC, and GmhB were found in the *S. sonnei* 4303 and *S. sonnei* Ss046 species. The *gmhD* gene, however, showed a frameshift mutation in the *S. sonnei* 4351, involving the deletion of two nucleotides at positions 806 and 807 (Figure 2). The mutation was validated by the Sanger sequencing method.

This mutation was studied further. Despite the mutation of the *gmhD* gene and the loss of an active GmhD protein, the expression of the *gmhD* gene increased compared to gene production in the wild-type bacterial strain, *S.* *sonnei* 4351. The whole transcriptome sequencing was validated by qPCR measurements in triplicate, showing the fold change to be 2.19, and the standard deviation of the measured cycle threshold was 0.15.

The thermosensitivity of the mutant strain was monitored with agar plate cultures and a much lower growth rate was detected in the case of *S. sonnei* 4351 above 42 °C. The increased susceptibility to erythromycin and cefalexin antibiotics was reflected by the MIC values. In the case of the *S. sonnei* 4351, the MIC was only 15.62 µg/mL, while a 125 µg/mL value was obtained with *S. sonnei* 4303 for erythromycin. The MIC for cefalexin was half for the *S. sonnei* 4351 (62.5 µg/mL) compared to that with *S. sonnei* 4303 (125 µg/mL). Much lower MIC values (1 µg/mL and 0.5 µg/mL) were obtained for the two aminoglycoside antibiotics, gentamicin and tobramycin, respectively, for both strains.

**Figure 3:**
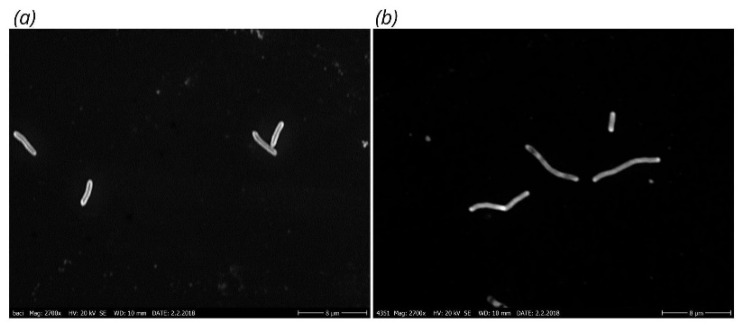
Scanning electron microscopic images of *S. sonnei* cells, showing (**a**) normal cell morphology for *Shigella sonnei* 4303 and (**b**) chains of 2–5 cells with septa for *Shigella sonnei* 4351. The magnification was 2700×.

The *S. sonnei* 4351 cells formed short chains with septa upon cell division (Figure 3). Chains with two or three (maximum five) cells were found with ca. 80% of the bacteria.

## 4. Discussion

### 4.1. Structural Consequence of Mutation in the Biosynthesis

**Figure 4:**
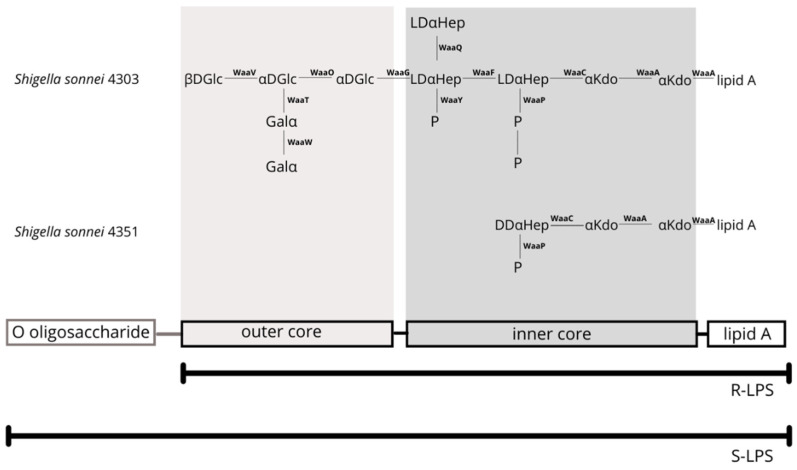
The structures of the R-type lipopolysaccharides from *S. sonnei* 4303 and *S. sonnei* 4351. No O-specific side chains are found in these bacteria. Genes involved in the LPS biosynthesis are indicated. The LPS in the *S. sonnei* 4351 strain contains a truncated core structure, due to the stop in the biosynthesis following the incorporation of a DDαHep.

Since the LPS structures of *S. sonnei* 4303 and 4351 are known (Figure 4), showing a well-defined R-type structure [27,32], the comparative genetic analysis makes them ideal subjects to perform further experiments on the hypothetic effect of heptose biosynthesis.

**Figure 5:**
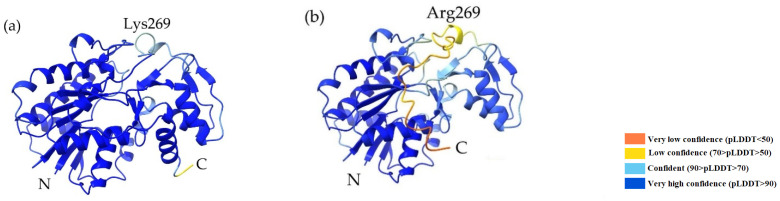
The 3D structures of the gene products of *gmhD* predicted by the AlphaFold (**a**) in *Shigella sonnei* 4303 and (**b**) in *Shigella sonnei* 4351. The colors show the confidence values of the prediction. The frameshift mutation causes the amino acid change at the 269th position. N and C mark the respective termini of the polypeptide chains.

GmhD works as an ADP-glycero-manno-heptose-6-epimerase. The AlphaFold prediction of the GmhD proteins from the sequences of the parent and the mutant strains are shown in Figure 5. Two domains have been distinguished in a previous study by Deacon et al. [17]: one is a NADP-binding domain that is a modified Rossmann fold with a central seven-stranded parallel β-sheet, flanked on either side by a total of seven α-helices; and the other is a smaller domain with three α-helices and two small parallel β-sheets, which defines the specificity for the substrate [43]. The predicted protein structure of the parent strain (Figure 5a) is fitting to the previous results; however, the frameshift mutation significantly affected the protein structure in the mutant bacterium (Figure 5b). The change in the polypeptide sequence occurs at the 269th position, which is lysine in the GmhD of *S. sonnei* 4303, and it is an arginine in *S. sonnei* 4351. The predicted 3D structures clearly show the low confidence of the highly disordered region at the C terminus of the mutant protein (Figure 5b).

The frameshift mutation in the *gmhD* sequence means that the epimerization at the C-6 position cannot occur, and this will result in the core of the lipopolysaccharide including only one D,D-heptose bound to the Kdo parts in *S. sonnei* 4351. The structural studies confirmed that only the L,D-heptoses makes it possible to elongate the lipopolysaccharides with the outer core, as shown in the case of *S. sonnei* 4303 in Figure 4 [44]. Since both enzymes, the heptosyltransferase I (Waac) and heptosyltransferase II (WaaF), which are responsible for the inclusion of the heptose sugars in the heptosyl-Kdo2-lipid A moiety, are stereoselective, the incorporation of a D,D-heptose in the structure will terminate further expansion of the molecule. In an in vitro study, Zamyatina et al. have discussed that the heptosyltransferase enzymes can use ADP-D-glycero-D-manno-heptose as a substrate; however, the reactions need at least tenfold higher concentrations in comparison with that of ADP-L-glycero-D-manno-heptose [43].

### 4.2. Role of Mutation in Bacterial Fitness

According to the literature, thermosensitivity of bacterial strains has been found in bacteria upon the mutation of genes, such as *gmhA*, *gmhB*, *gmhC*, *gmhD*, *waaC*, *waaF*, and *waaG* involved in the heptose biosynthesis, or in the transfer of LPS core constituents [45,46]. Our experiments also showed a decreased proliferation rate of the *S. sonnei* 4351 strain at a higher temperature, showing the probable connection between thermosensitivity and *gmhD* activity [45]. This effect could be suppressed by adding Mg^2+^ to the culture medium, suggesting a connection between thermosensitivity and decreased outer membrane stability.

The increased susceptibility against polymyxins has been known previously; however, our results suggest that due to the mutation in the *gmhD* gene, an increased susceptibility was achieved against the macrolide and cephalosporin antibiotics as well. A targeting of this gene may be of therapeutic relevance.

Considering the aberrant cell division and the above-mentioned changes in the bacterial fitness, the results confirm the pleiotropic effect of *gmhD* mutation, showing how this single mutation may lead to more drastic consequences beyond the heptose biosynthesis. The inner core of the endotoxins plays a critical role in the stability of the outer membrane, since the structure of this section is more conserved, making it a good general target on Gram-negative bacteria. Bacteria with no O side-chain or lacking the core oligosaccharide side chain are viable; however, the absence of these molecules changes the general features of the microorganism. The truncated lipopolysaccharides are known to initiate mucoid phenotype and enhanced binding effectivity to antimicrobial chemokines, too [47]; however, attempts to influence LPS biosynthesis were not successful, so far [48].

The results show the high significance of the GmhD in bacterial function beyond lipopolysaccharide core biosynthesis and suggest further investigation, as a target, in the fight against Gram-negative bacterial infections.

## Figures and Tables

**Table 1:** Primer and probe sequences used to amplify *uidA* and *gmhD* sequences for qPCR analyses.

*uidA*	
Forward primer	GAATACGGCGTGGATACGTTAG (sense)
Reverse primer	GATCAAAGACGCGGTGATACA (antisense)
Probe	TGAAGAGTATCAGTGTGCATGGCTGG (sense)
*gmhD*	
Forward primer	CGTTGAACGTCTACGGTTACTC (sense)
Reverse primer	CCTTCACGCGGTCCATAAA (antisense)
Probe	TCGCAGATTGTTGGCTTCCGCTAT (sense)

**Table 2:** Primer sequences used to amplify *gmhD* sequences for Sanger sequencing.

*gmhD*	
Forward primer	ATGATCATCGTTACCGGCGGC
Reverse primer	TTATGCGTCGCGATTCAGCCA

## Data Availability

The genome sequence of *S. sonnei* 4351 was deposited in GenBank under the accession number PRJNA400697.

## References

[1] Meredith T.C., Aggarwal P., Mamat U., Lindner B., Woodard R.W. (2006). Redefining the Requisite Lipopolysaccharide Structure in *Escherichia coli*. ACS Chem. Biol..

[2] Anderson M., Bull H., Galloway S., Kelly T., Mohan S., Radika K., Raetz C. (1993). UDP-N-acetylglucosamine acyltransferase of *Escherichia coli*. The first step of endotoxin biosynthesis is thermodynamically unfavorable. J. Biol. Chem..

[3] Sorensen P.G., Lutkenhaus J., Young K., Eveland S.S., Anderson M.S., Raetz C.R. (1996). Regulation of UDP-3-O-[R-3-hydroxymyristoyl]-N-acetylglucosamine Deacetylase in *Escherichia coli*: The second enzymatic step of lipid a biosynthesis. J. Biol. Chem..

[4] Bartling C.M., Raetz C.R.H. (2008). Steady-State Kinetics and Mechanism of LpxD, the N-Acyltransferase of Lipid A Biosynthesis. Biochemistry.

[5] Babinski K.J., Ribeiro A.A., Raetz C.R.H. (2002). The *Escherichia coli* Gene Encoding the UDP-2,3-diacylglucosamine Pyrophosphatase of Lipid A Biosynthesis. J. Biol. Chem..

[6] Radika K., Raetz C.R. (1988). Purification and properties of lipid A disaccharide synthase of *Escherichia coli*. J. Biol. Chem..

[7] Garrett T.A., Que N.L., Raetz C.R. (1998). Accumulation of a Lipid A Precursor Lacking the 4′-Phosphate following Inactivation of the *Escherichia coli* lpxKGene. J. Biol. Chem..

[8] Sperandeo P., Pozzi C., Dehò G., Polissi A. (2006). Non-essential KDO biosynthesis and new essential cell envelope biogenesis genes in the *Escherichia coli* yrbG–yhbG locus. Res. Microbiol..

[9] Rick P.D., Osborn M.J. (1977). Lipid A mutants of *Salmonella typhimurium*. Characterization of a conditional lethal mutant in 3-deoxy-D-mannooctulosonate-8-phosphate synthetase. J. Biol. Chem..

[10] Ray P.H., Benedict C.D. (1980). Purification and Characterization of a Specific 3-Deoxy-D-manno-Octulosonate 8-Phosphate Phosphatase from *Escherichia coli* B. J. Bacteriol..

[11] Heyes D., Levy C., Lafite P., Roberts I.S., Goldrick M., Stachulski A.V., Rossington S.B., Stanford D., Rigby S.E.J., Scrutton N.S. (2009). Structure-based mechanism of CMP-2-keto-3-deoxymanno-octulonic acid synthetase: Convergent evolution of a sug-ar-activating enzyme with DNA/RNA polymerases. J. Biol. Chem..

[12] Belunis C.J., Clementz T., Carty S.M., Raetz C.R.H. (1995). Inhibition of Lipopolysaccharide Biosynthesis and Cell Growth following Inactivation of the kdtA Gene in *Escherichia coli*. J. Biol. Chem..

[13] Somerville J.E., Cassiano L., Bainbridge B., Cunningham M.D., Darveau R.P. (1996). A novel *Escherichia coli* lipid A mutant that produces an antiinflammatory lipopolysaccharide. J. Clin. Investig..

[14] Kneidinger B., Marolda C., Graninger M., Zamyatina A., McArthur F., Kosma P., Valvano M.A., Messner P. (2002). Biosynthesis pathway of ADP-L-glycero-β-D-manno-heptose in *Escherichia coli*. J. Bacteriol..

[15] Taylor P.L., Blakely K.M., de León G.P.-P., Walker J.R., McArthur F., Evdokimova E., Zhang K., Valvano M., Wright G., Junop M.S. (2008). Structure and Function of Sedoheptulose-7-phosphate Isomerase, a Critical Enzyme for Lipopolysaccharide Biosynthesis and a Target for Antibiotic Adjuvants. J. Biol. Chem..

[16] Güzlek H., Graziani A., Kosma P. (2005). A short synthesis of D-glycero-D-manno-heptose 7-phosphate. Carbohydr. Res..

[17] Deacon A., Ni Y., Coleman W., Ealick S. (2000). The crystal structure of ADP-L-glycero-D-mannoheptose 6-epimerase: Catalysis with a twist. Structure.

[18] Kadrmas J.L., Raetz C.R.H. (1998). Enzymatic Synthesis of Lipopolysaccharide in *Escherichia coli*. Purification and Properties of Hepto-Syltransferase I. J. Biol. Chem..

[19] Nikaido H. (1976). Outer membrane of *Salmonella typhimurium*: Transmembrane diffusion of some hydrophobic substances. Biochim. Biophys. Acta Biomembr..

[20] Raetz C.R.H., Whitfield C. (2002). Lipopolysaccharide Endotoxins. Annu. Rev. Biochem..

[21] Onishi H.R., Pelak B.A., Gerckens L.S., Silver L.L., Kahan F.M., Chen M.-H., Patchett A.A., Galloway S.M., Hyland S.A., Anderson M.S. (1996). Antibacterial Agents That Inhibit Lipid A Biosynthesis. Science.

[22] Mdluli K.E., Witte P.R., Kline T., Barb A.W., Erwin A.L., Mansfield B.E., McClerren A.L., Pirrung M.C., Tumey L.N., Warrener P. (2006). Molecular Validation of LpxC as an Antibacterial Drug Target in *Pseudomonas aeruginosa*. Antimicrob. Agents Chemother..

[23] Pfannkuch L., Hurwitz R., Trauisen J., Sigulla J., Poeschke M., Matzner L., Kosma P., Schmid M., Meyer T.F. (2019). ADP heptose, a novel pathogen-associated molecular pattern identified in *Helicobacter pylori*. FASEB J..

[24] Posch G., Andrukhov O., Vinogradov E., Lindner B., Messner P., Holst O., Schäffer C. (2013). Structure and Immunogenicity of the Rough-Type Lipopolysaccharide from the Periodontal Pathogen *Tannerella forsythia*. Clin. Vaccine Immunol..

[25] Ledov V.A., Golovina M.E., Markina A.A., Knirel Y.A., L’Vov V.L., Kovalchuk A.L., Aparin P.G. (2019). Highly homogenous tri-acylated S-LPS acts as a novel clinically applicable vaccine against *Shigella flexneri* 2a infection. Vaccine.

[26] Goyette-Desjardins G., Auger J.-P., Dolbec D., Vinogradov E., Okura M., Takamatsu D., Van Calsteren M.-R., Gottschalk M., Segura M. (2020). Comparative study of immunogenic properties of purified capsular polysaccharides from *Streptococcus suis* serotypes 3, 7, 8, and 9: The serotype 3 polysaccharide induces an opsonizing IgG response. Infect. Immun..

[27] Bui A., Kilár A., Dörnyei Á., Poór V., Kovács K., Kocsis B., Kilár F. (2011). Carbohydrate composition of endotoxins from R-type isogenic mutants of *Shigella sonnei* studied by capillary electrophoresis and GC-MS. Croat. Chem. Acta.

[28] Rauss K., Kétyi I., Vertényi A., Vörös S. (1954). Studies on the nature of phase variation of *Shigella sonnei*. Acta microbiol. Acad. Sci. Hung..

[29] Kocsis T.B., Kontrohr V., László H. (1980). Milch, Biosynthesis of the cell-wall of *Shigella sonnei*. 1. Isolation and characterization of different defective mutants. Acta Microbiol. Acad. Sci. Hung..

[30] Kontrohr T., Kocsis B. (1981). Isolation of adenosine 5’-diphosphate-D-glycero-D-mannoheptose. An intermediate in lipopolysaccharide biosynthesis of *Shigella sonnei*. J. Biol. Chem..

[31] Kocsis B., Kontrohr T. (1984). Isolation of adenosine 5’-diphosphate-L-glycero-D-mannoheptose, the assumed substrate of heptose transferase(s), from *Salmonella minnesota* R595 and *Shigella sonnei* Re mutants. J. Biol. Chem..

[32] Makszin L., Kilár A., Felső P., Péterfi Z., Kocsis B., Kilár F. (2012). Quantitative microfluidic analysis of S- and R-type endotoxin components with chip capillary electrophoresis. Electrophoresis.

[33] Raetz C.R.H. (1990). Biochemistry of endotoxins. Annu. Rev. Biochem..

[34] Deutsch-Nagy L., Urbán P., Tóth Z., Bihari Z., Kocsis B., Fekete C., Kilár F. (2018). Genome sequence of *Shigella sonnei* 4303. Gut Pathog..

[35] Kanehisa M., Goto S. (2000). KEGG: Kyoto Encyclopedia of Genes and Genomes. Nucleic Acids Res..

[36] Bankevich A., Nurk S., Antipov D., Gurevich A.A., Dvorkin M., Kulikov A.S., Lesin V.M., Nikolenko S.I., Pham S., Prjibelski A.D. (2012). SPAdes: A new genome assembly algorithm and its applications to single-cell sequencing. J. Comput. Biol..

[37] Darling A.C.E., Mau B., Blattner F.R., Perna N.T. (2004). Mauve: Multiple Alignment of Conserved Genomic Sequence with Rearrangements. Genome Res..

[38] Seemann T. (2014). Prokka: Rapid Prokaryotic Genome Annotation. Bioinformatics.

[39] GenBank Data-National Center for Biotechnology Information. https://www.ncbi.nlm.nih.gov/assembly/GCA_002811105.1/.

[40] Altschul S.F., Gish W., Miller W., Myers E.W., Lipman D.J. (1990). Basic local alignment search tool. J. Mol. Biol..

[41] Vaara M. (1990). Antimicrobial susceptibility of *Salmonella typhimurium* carrying the outer membrane permeability mutation SS-B. Antimicrob. Agents Chemother..

[42] Jumper J., Evans R., Pritzel A., Green T., Figurnov M., Ronneberger O., Tunyasuvunakool K., Bates R., Žídek A., Potapenko A. (2021). Highly accurate protein structure prediction with AlphaFold. Nature.

[43] Zamyatina A., Gronow S., Puchberger M., Graziani A., Hofinger A., Kosma P. (2003). Efficient chemical synthesis of both anomers of ADP L-glycero- and D-glycero-D-manno-heptopyranose. Carbohydr. Res..

[44] Kilár A., Dörnyei A., Bui A., Szabó Z., Kocsis B., Kilár F. (2010). Structural variability of endotoxins from R-type isogenic mutants of *Shigella sonnei*. Biol. Mass Spectrom..

[45] Karow M., Raina S., Georgopoulos C., Fayet O. (1991). Complex phenotypes of null mutations in the htr genes, whose products are essential for *Escherichia coli* growth at elevated temperatures. Res. Microbiol..

[46] Murata M., Fujimoto H., Nishimura K., Charoensuk K., Nagamitsu H., Raina S., Kosaka T., Oshima T., Ogasawara N., Yamada M. (2011). Molecular strategy for survival at a critical high temperature in *Eschierichia coli*. PLoS ONE.

[47] Erickson D.L., Lew C.S., Kartchner B., Porter N., McDaniel S.W., Jones N.M., Mason S., Wu E., Wilson E. (2016). Lipopolysaccharide biosynthesis genes of *Yersinia pseudotuberculosis* promote resistance to antimicrobial chemokines. PLoS ONE.

[48] Deutsch-Nagy L., Urbán P., Szebeni H., Albert B., Kocsis B., Kilár F. (2019). Closantel as a potential lipopolysaccharide biosynthesis inhibitor in *Shigella sonnei* 4303. Stud. Univ. Babes Bolyai Chem..

